# Factors associated with changes in adequate antenatal care visits among pregnant women aged 15-49 years in Tanzania from 2004 to 2016

**DOI:** 10.1186/s12884-021-04350-y

**Published:** 2022-01-07

**Authors:** Elizabeth Kasagama, Jim Todd, Jenny Renju

**Affiliations:** 1 Department of Epidemiology and Biostatistics, Institute of Public Health, Kilimanjaro Christian Medical University College (KCMUCo), P.O Box 2240, Kilimanjaro, Tanzania; 2 London School of Hygiene and Tropical Medicine (LSTM), London, UK

**Keywords:** Antenatal care, Adequate ANC, Changes, Maternal health, Maternal mortality, Tanzania

## Abstract

**Background:**

Antenatal care (ANC) is crucial for the health of the mother and unborn child as it delivers highly effective health interventions that can prevent maternal and newborn morbidity and mortality. In 2002, the World Health Organization (WHO) recommended a minimum of four ANC visits for a pregnant woman with a positive pregnancy during the entire gestational period. Tanzania has sub-optimal adequate (four or more) ANC visits, and the trend has been fluctuating over time. An understanding of the factors that have been contributing to the fluctuating trend over years is pivotal in increasing the proportions of pregnant women attaining adequate ANC visits in Tanzania.

**Methods:**

The study used secondary data from Tanzania Demographic Health Survey (TDHS) from 2004 to 2016. The study included 17976 women aged 15-49 years. Data were analyzed using Stata version 14. Categorical and continuous variables were summarized using descriptive statistics and weighted proportions. A Poisson regression analysis was done to determine factors associated with adequate ANC visits. To determine factors associated with changes in adequate ANC visits among pregnant women in Tanzania from 2004 to 2016, multivariable Poisson decomposition analysis was done.

**Results:**

The overall proportion of women who had adequate ANC visits in 2004/05, 2010 and 2015/16 was 62, 43 and 51% respectively. The increase in the proportion of women attaining adequate ANC from 2010 to 2015/16 was mainly, 66.2% due to changes in population structure, thus an improvement in health behavior. While 33.8% was due to changes in the mother’s characteristics. Early initiation of first ANC visit had contributed 51% of the overall changes in adequate ANC attendance in TDHS 2015/16 survey.

**Conclusion:**

Early ANC initiation has greatly contributed to the increased proportion of pregnant women who attain four or more ANC visits overtime. Interventions on initiating the first ANC visit within the first twelve weeks of pregnancy should be a priority to increase proportion of women with adequate ANC visit.

## Background

Adequate and quality antenatal care (ANC) is effective at promoting better health outcomes for both mother and child during pregnancy [1, 2]. Strong evidence exists to support the link between ANC during pregnancy, skilled birth attendants during delivery, and quality postnatal care and reduced maternal and infant morbidity and mortality [3–8]. Globally, almost 60% of stillbirths are due to poor fetal growth, untreated and unattended maternal infection, and conditions that could have been avoided or treated by expert attention during ANC visits [7]. A wide range of services can be offered during ANC including screening, detection, prevention and treatment of any pregnancy-related complication, infection or morbidity [9].

The WHO 2002 Focus Antenatal Care (FANC) model recommends a minimum of four ANC visits for a woman with an uncomplicated pregnancy, with the first visit occurring during the first twelve weeks of pregnancy, although currently there is an 8-contact model in place [9, 10]. In 2002, Tanzania adopted FANC, however, the first ANC is to be initiated within 16 gestational weeks [11]. Globally, ANC coverage (at least one visit during pregnancy) is 86%, yet only 62% of women meet the recommended four ANC visits. In Africa, ANC coverage is 69% with only 54% of women attending the minimum of four ANC visits, while in Tanzania, ANC coverage is higher, 98% but only 51% of pregnant women attain the minimum of four ANC visits [12]. Despite high ANC coverage, adequate (four or more) ANC visits are still suboptimal and could partly explain the unacceptably high neonatal mortality and stillbirth rates in Tanzania, with 25 deaths/1000 live births and 39 deaths/1000 pregnancies, respectively [13].

To scale up the uptake of ANC and to address the burden of maternal mortality, additional interventions were introduced. These included Exemptions from paying user fees on health care services for maternal, newborn and child under five. In 2012, Tanzania’s National Safe Motherhood Campaign (Wazazi Nipendeni) was implemented to encourage pregnant women to initiate the first ANC within 12 weeks of pregnancy and adhere to ANC services [14]. In 2014, the “Big Results Now” and the “Sharpened One Plan” programs were implemented. Despite these interventions, the Tanzania Demographic and Health Survey (TDHS) reported a fluctuating trend in adequate ANC visits: a fall from 62 to 43% and a rise to 51% for 2004/05, 2010 and 2015/16 surveys, respectively [13]. This suboptimal ANC attendance has been associated with a several factors, as documented in various studies worldwide. The factors include but are not limited to: long distance to a health facility, geographical zone, first ANC initiation, woman’s desire to avoid pregnancy, marital status, wealth quintiles, multiparity, living in an urban area, and higher education level [14–19].

This study aimed to show how much each individual factor has contributed to the decline from 2004/04 to 2010 and the increase from 2010 to 2015/16 in the TDHS and how they have contributed to the varying low proportions of adequate ANC visits over time. Filling this knowledge gap may help identify the key contributing factors and provide valuable information on how the programmatic changes during the 2004–2016 period have impacted adequate ANC attendance. Moreover, understanding the factors associated with changes in adequate ANC visits may help to provide useful information to policymakers, project implementing partners and in designing target interventions that may improve adequate ANC visits in Tanzania.

## Methods

### Study design and study settings

The study was conducted in Tanzania, which includes the mainland and island. This was a Crossectional study that used data from the Tanzania Demographic Health Survey (TDHS), Further details of the survey are available elsewhere [13], but in brief this is a national representative survey done after five years with the objective to obtain the current and reliable information on demographic and health indicators about family planning, fertility levels and preferences, maternal mortality, infant and child mortality, nutritional status of mothers and children, ANC, delivery care, and childhood immunizations and diseases. Data were obtained from www.dhsprogram.com, after being granted permission to access and use TDHS data. Data from 2004/04, 2010 and 2015/16 surveys were used.

### Study population

**Fig. 1:**
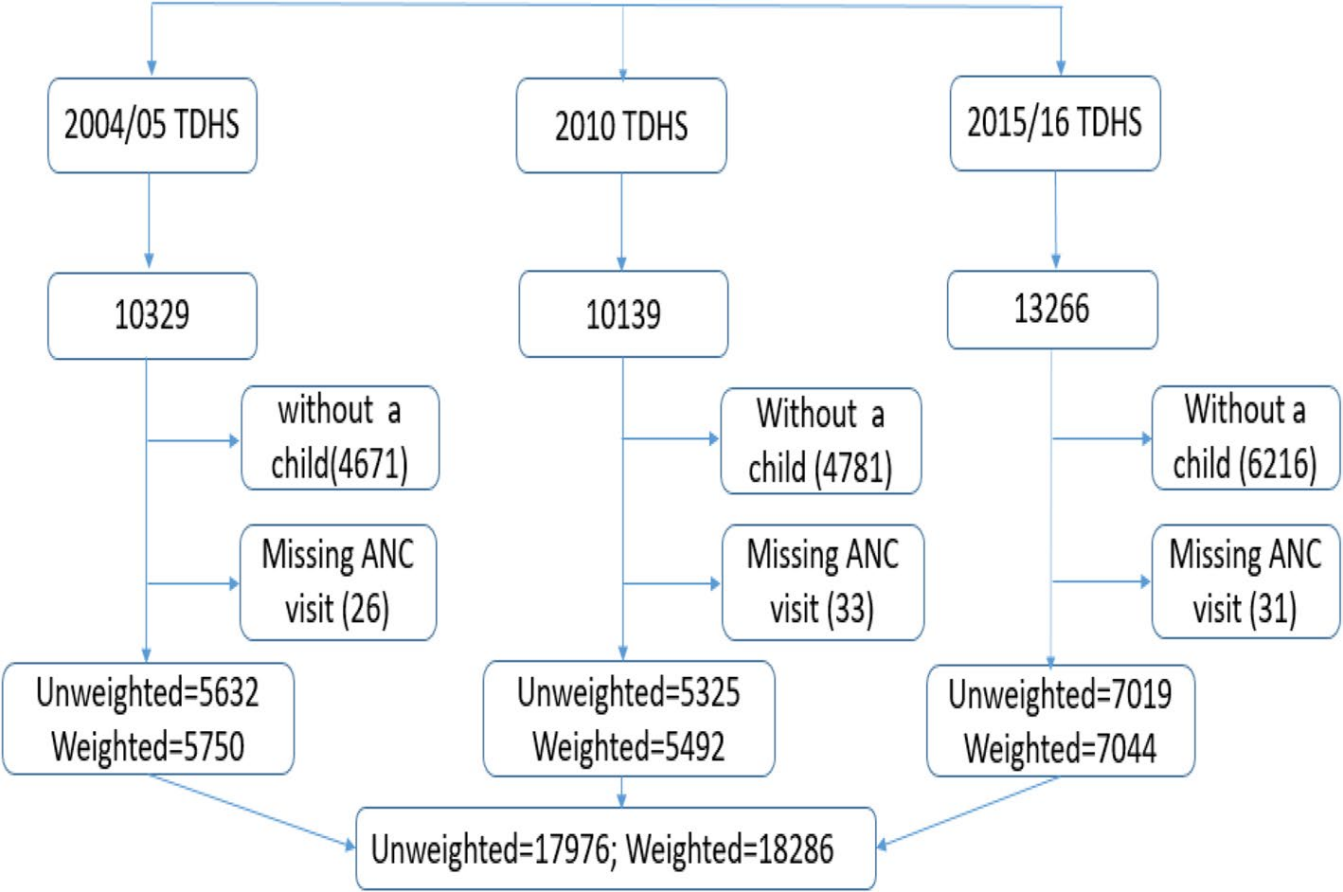
Flow chart showing participants enrolled in the study per respective survey years

The population was all women of reproductive age (15-49 years) who had given birth to at least one child within the five years before the survey and had information on ANC visits. For a woman with multiple births during the five-year period, we considered mother’s last birth within 5 years prior the survey for this analysis. A total of 33,734 women aged 15-49 years in Tanzania participated in the three TDHS surveys. After excluding those with missing information on ANC visits, we remained with a total of 17,976. Of 17,976 women enrolled in the study: 4541(77.9%), 4201(76.9%) and 5193(70.1%) for 2004/05, 2010 and 2015/16 surveys respectively (Fig. 1).

### Study variables

Our dependent variable was adequate ANC visits, which was categorized as four or more ANC visits and coded 1, less than four ANC visits as inadequate were coded 0. Independent variables were respondent’s age at last birth (15-19 years, 20-24 years, 25-29 years, 30-34 years, 35+ years), education level (no formal education, primary education, secondary and higher education), employment status (unemployed, employed), marital status (married/cohabiting, single, divorced/widowed/separated), residence (urban, rural), wealth index (poorest, poorer, middle, richer, richest), zones; these are administrative regions grouped according to geographical location (western zone, northern zone, central zone, southern highlands, southern zone, south west highlands zone, lake zone, eastern zone, Zanzibar), first ANC initiated (women with first ANC visit later than 12 gestational weeks, women with first ANC visit by 12 gestational weeks), decision maker of respondent’s health care (respondent alone, respondent and partner, partner alone, someone else), parity (1 child, 2-3 children, 4-5 children, 6 or more children), frequency of listening to radio (not at all, Less than once a week, at least once a week), frequency of watching TV (not at all, less than once a week, at least once a week), desire of last pregnancy (wanted then, wanted later, wanted no more), history of terminated pregnancy (never had, ever had) and distance from health facility (big problem, not a big problem). The selection of variables was made using the Andersen’s Behavioural Model of Health Services Use [20]. All these variables were considered as mother’s characteristics and population characteristics in the analysis.

### Statistical analysis

Data were analyzed using STATA Corporation, College Station, TX, USA version 14 (Stata/SE 14.2). The analysis considered the complex survey features: primary sampling units, strata, and sampling weights. A Poisson regression analysis was done to determine factors associated with adequate ANC visits. Multivariable Poisson decomposition analysis was conducted to determine factors associated with changes in adequate ANC visits. Decomposition analysis was conducted to understand whether observed changes in adequate ANC visits could be explained by changes in factors over time or in the population structure (population dynamics). To explain the observed change in the percentage of pregnant women attaining adequate ANC visits, we used the Blinder-Oaxaca decomposition analysis [21–23]. The main goal decomposition analysis was to explain on the individual contributions of the factors on adequate ANC visits differences among pregnant women in Tanzania in different surveys. The differentials in adequate ANC visits between these groups was portioned into two components, one that can be attributable to differences in characteristics and the component that is attributable to the effect of those characteristics. The factors might have a different contribution on the change observed at different survey period. The decomposition analysis was done between two time points, at first, we decomposed survey year 2004/05 to 2010 and lastly survey year 2015/16 to 2010. The baseline survey year was the one with the lowest proportion of pregnant women with adequate ANC visits, thus survey year 2010 for both decomposition analysis. Contributions were considered statistically significant at a *P*-value of less than 0.05.

## Results

### Characteristics of the study participants

A total of 17,976 women were included in the analysis. Most of the participants were from rural areas, the mean age (±SD) of the study population was 27.06 (±7.00). More than half of the respondents in each survey had at least primary education level. Most of the participants were married or cohabiting: 85.4, 84.2 and 81.6% for 2004/05, 2010 and 2015/16 survey, respectively. The proportions of women aged 35 to 45 years increased across the survey years, from 15.6%, in 2004/05 to 17.8% in 2015/16. The percentage of women who achieved secondary education and above also increased from 9.2% in 2004/05 to 19.9% in 2015/16 and the percentage of women without formal education decreased from 26.8% in 2004/05 to 19.5% in 2015/16. Substantial regional variation in survey participation was observed; throughout the three surveys, the Lake Zone had the highest percentage of women participating in the survey while the Southern zone had lowest (Table 1).

**Table 1:** Characteristics of the study participants (*N* = 17976)

Variables	2004-05 TDHS	2010 TDHS	2015-16 TDHS
	Frequency (%) (***n*** = 5632)	Frequency (%) (***n*** = 5325)	Frequency (%) (***n*** = 7019)
***Age at delivery (in years)***			
15-19	961(17.1)	775(14.5)	1253(17.8)
20-24	1532(27.2)	1376(25.8)	1733(24.7)
25-29	1326(23.5)	1249(23.5)	1593(22.7)
30-34	933(16.6)	940(17.7)	1194(17.0)
35+	880(15.6)	985(18.5)	1246(17.8)
Mean age (±SD)	27.1(±7.07)	27.80(±7.10)	27.41(±7.23)
***Zones***			
Western zone	522(9.3)	502(9.4)	619(8.8)
Northern zone	546(9.7)	489(9.2)	562(8.0)
Central zone	676(12.0)	625(11.7)	690(9.8)
Southern Highlands	399(7.1)	366(6.9)	561(8.0)
Southern zone	368(6.5)	342(6.4)	347(4.9)
Southwest Highlands zone	499(8.9)	410(7.7)	778(11.1)
Lake Zone	1051(18.7)	1020(19.2)	1803(25.7)
Eastern zone	522(9.3)	534(10.0)	713(10.2)
Zanzibar	1049(18.5)	1037(19.5)	946(13.5)
***Place of residence***			
Rural	4541(80.6)	4201(78.9)	5193(74.0)
Urban	1091(19.4)	1124(21.1)	1826(26.0)
***Highest level of education***			
No formal education	1509(26.8)	1262(23.7)	1368(19.5)
Primary education	3603(64.0)	3393(63.7)	4255(60.6)
Secondary and above	520(9.2)	670(12.6)	1396(19.9)
***Current marital status***			
Single	285(5.0)	296(5.6)	451(6.4)
Married/Cohabiting	4809(85.4)	4486(84.2)	5724(81.6)
Widowed/Divorced/Separated	538(9.6)	543(10.2)	844(12.0)
***Employment status***^ab^			
Unemployed	1029(18.3)	929(17.5)	1515(21.6)
Employed	4602(81.7)	4388(82.5)	5504(78.4)
***Wealth index***			
Poorest	1167(20.7)	1006(18.9)	1441(20.5)
Poorer	1131(20.1)	1150(21.6)	1356(19.3)
Middle	1082(19.2)	1078(20.2)	1376(19.6)
Richer	1247(22.1)	1161(21.8)	1544(22.0)
Richest	1005(17.9)	930(17.5)	1302(18.6)

^a^ **Employment status 2004/5 (*n*** **= 5631)**

^b^ **Employment status 2010 (*n*** **= 5317)**

### Trends of adequate antenatal care visits

**Fig. 2:**
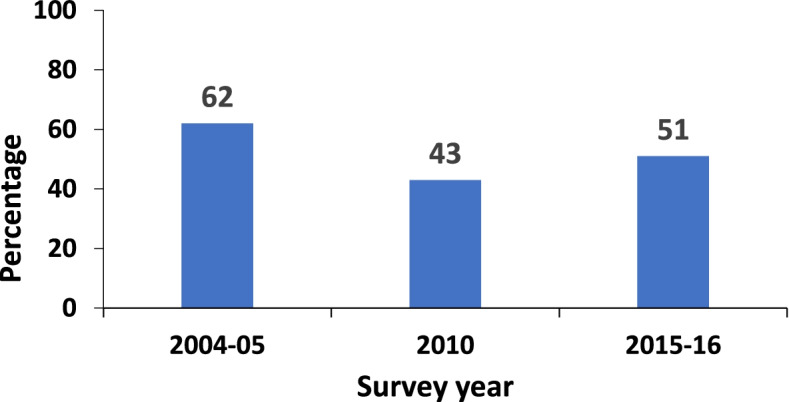
Percentage of pregnant women with adequate ANC visits from 2004 to 2016

**Fig. 3:**
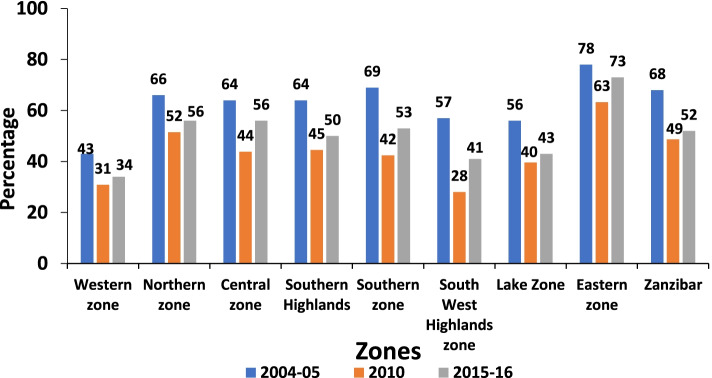
Percentage of pregnant women with adequate ANC visits by zones in Tanzania from 2004 to 2016

**Fig. 4:**
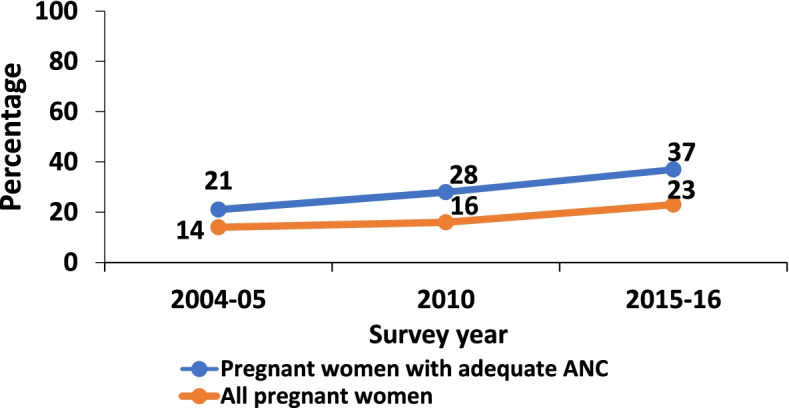
Percentage of pregnant women with first ANC visit in first trimester from 2004 to 2016

The trend in adequate ANC attendance has fluctuated over time. Adequate ANC attendance decreased from 61% in 2004/05 to 43% in the 2010 survey and then increased again to 51% in the 2015/16 survey (Fig. 2). A similar pattern was also found when stratified by geographical zone. The eastern zone had the highest percentage of women with adequate ANC attendance for all three surveys (Fig. 3). The percentage of women with four ANC visits who initiated their first ANC visit in the first trimester increased over time (Fig. 4).

### Factors associated with adequate antenatal care visits

Various factors were associated with adequate ANC visits for each survey, including first ANC in the first trimester, multiparity, wanting pregnancy later, watching TV at least once a week, older age, geographical zone, secondary education and above, reasonable distance to a health facility, richer and richest household wealth index (Table 2).

**Table 2:** Factors associated with adequate antenatal care visits in Tanzania

Characteristics	2004-05 TDHS	2010 TDHS	2015-16 TDHS
	APR (95% CI)	APR (95% CI)	APR (95% CI)
***Age at delivery (in years)***			
15-19	1	1	1
20-24	0.92(0.85-0.99)	1.21(1.05-1.40)	1.06(0.96-1.18)
25-29	0.95(0.87-1.04)	1.21(1.03-1.42)	1.04(0.93-1.17)
30-34	0.95(0.85-1.06)	1.45(1.20-1.74)	1.15(1.01-1.30)
35+	0.96(0.85-1.09)	1.28(1.03-1.58)	1.19(1.02-1.38)
***Zones***			
Western zone	1	1	1
Northern zone	1.47(1.31-1.66)	1.45(1.19-1.77)	1.52(1.31-1.76)
Central zone	1.50(1.34-1.69)	1.37(1.13-1.66)	1.71(1.48-1.98)
Southern Highlands	1.37(1.21-1.56)	1.11(0.89-1.38)	1.25(1.07-1.46)
Southern zone	1.49(1.32-1.69)	1.16(0.93-1.45)	1.30(1.10-1.54)
Southwest Highlands	1.40(1.23-1.58)	0.74(0.58-0.96)	1.25(1.05-1.49)
Lake Zone	1.29(1.15-1.44)	1.27(1.06-1.53)	1.47(1.28-1.68)
Eastern zone	1.62(1.44-1.81)	1.53(1.26-1.85)	1.71(1.49-1.97)
Zanzibar	1.46(1.31-1.63)	1.27(1.05-1.53)	1.48(1.28-1.72)
***Place of residence***			
Rural	1.00(0.94-1.08)	0.98(0.87-1.11)	0.93(0.85-1.01)
Urban	1	1	1
***Highest level of education***			
No formal education	1	1	1
Primary education	1.04(0.98-1.10)	1.13(1.01-1.26)	1.05(0.96-1.14)
Secondary and above	1.09(0.99-1.20)	1.25(1.06-1.48)	1.12(1.01-1.25)
***Parity***			
1	1	1	1
2-3	0.98(0.92-1.05)	0.89(0.78-1.02)	0.88(0.81-0.96
4-5	0.99(0.92-1.06)	0.76(0.64-0.91)	0.83(0.74-0.94)
6+	0.95(0.87-1.03)	0.74(0.60-0.92)	0.78(0.67-0.91)
***Decision maker on respondent’s health***			
Respondent alone	1	1	1
Respondent and partner	0.94(0.88-1.00)	0.93(0.83-1.03)	1.05(0.97-1.13)
Partner alone	0.96(0.91-1.01)	0.87(0.78-0.98)	0.93(0.87-1.03)
Someone else	0.89(0.80-0.99)	0.89(0.58-1.37)	1.02(0.62-1.68)
***Desire of last pregnancy***			
Wanted then	1	1	1
Wanted later	0.96(0.90-1.02)	0.82(0.74-0.92)	0.92(0.86-0.98)
Wanted no more	0.95(0.86-1.06)	1.06(0.85-1.31)	0.93(0.78-1.11)
***Wealth index***			
Poorest	1	1	1
Poorer	1.08(1.00-1.17)	0.95(0.83-1.09)	1.03(0.93-1.14)
Middle	1.07(0.99-1.16)	1.03(0.90-1.17)	1.05(0.95-1.16)
Richer	1.12(1.03-1.21)	1.18(1.03-1.35)	1.22(1.11-1.35)
Richest	1.16(1.05-1.27)	1.10(0.93-1.31)	1.16(1.02-1.31)
***First ANC initiated***			
Later than 1^st^ trimester	1	1	1
Within 1^st^ trimester	1.47(1.41-1.52)	1.96(1.82-2.11)	1.89(1.79-2.00)
***Distance to Health facility***			
Not a big problem	1.06(1.01-1.12)	1.03(0.93-1.14)	1.05(0.99-1.11)
Big problem	1	1	1
***Frequency of watching TV***			
Not at all	1	1	1
Less than once a week	1.04(0.97-1.12)	1.03(0.91-1.17)	0.99(0.92-1.08)
At least once a week	1.01(0.93-1.08)	1.24(1.09-1.42)	1.05(0.96-1.16)

In the multivariable Poisson regression analysis, for all three surveys ANC initiation within the first trimester had a positive effect on adequate ANC visits. The proportion of women with adequate ANC attendance was 1.47 (95% CI: 1.41-1.52) times greater among women who initiated ANC within the first trimester compared to those who initiated later in the 2004/05 survey, 1.96 (95% CI: 1.82-2.11) times higher in 2010, and 1.89 (95% CI: 1.79-2.00) times higher in 2015/16. However, wanting pregnancy later had a negative influence on adequate ANC visits. In the 2004/05 survey, adequate ANC attendance was 0.96 (95% CI: 0.90-1.02) times lower among women who wanted pregnancy later compared to those who wanted pregnancy at that time, 0.82 (95% CI: 0.74-0.92) times lower in 2010, and 0.92 (95% CI: 0.86-0.98) times lower in 2015/16 (Table 2).

### Factors associated with changes in adequate antenatal care visits across the surveys

The multivariable decomposition regression models found that 95.8% of the decline in adequate ANC visits from 2004/05 (62%) to 2010 (43%) were attributed by changes in the coefficients (mother’s characteristics) and only 4.2% of the decline was due to changes in the population characteristics (population dynamics). There were no significant changes in the population structures during this period, suggesting that the population remained relatively static between the 2004/05 and 2010 surveys. The southwest highland zone contributed 14.2% to the observed decline in the 2004/05 and 2010 surveys, which was statistically significant. It means that the zone where a pregnant woman lived affected her ability to attain adequate ANC. Changes in the initiation of the first ANC within the first trimester slowed the decline by 8.7%, which was also statistically significant (Table 3).

**Table 3:** Decomposition of changes in adequate antenatal care visits 2004 to 2010

Characteristics	Differences in Population structure (E)			Differences in coefficients(C)		
	Coefficient	%	***p***-value	Coefficient	%	***p***-value
***Age at delivery (in years)***						
15-19	1.0			1.0		
20-24	0.0015	-0.7	0.352	0.0345	-16.3	0.001
25-29	-0.0007	-0.3	0.709	0.0232	-11.1	0.013
30-34	-0.0026	1.2	0.707	0.0284	-13.4	<0.001
35+	-0.0038	1.8	0.707	0.0148	-7	0.065
***Zones***						
Western zone	1.0			1.0		
Northern zone	-0.0035	1.7	0.711	-0.0008	0.4	0.865
Central zone	-0.0012	0.6	0.711	-0.0045	2.1	0.431
Southern Highlands	-0.0001	0.1	0.73	-0.0074	3.5	0.083
Southern zone	0.0002	-0.1	0.729	-0.0071	3.3	0.037
Southwest Highlands	-0.0008	0.4	0.71	-0.0301	14.2	<0.001
Lake Zone	0.0033	-1.6	0.713	-0.0014	0.7	0.941
Eastern zone	0.0029	-1.4	0.711	-0.0042	2	0.551
Zanzibar	-0.0005	0.2	0.713	-0.0014	0.7	0.291
***Highest level of education***						
No formal education	1.0			1.0		
Primary education	0.0014	-0.7	0.713	0.0197	-9.3	0.34
Secondary and above	-0.0014	0.7	0.709	0.002	-0.9	0.448
***Parity***						
1 child	1.0			1.0		
2-3 children	-0.0004	0.2	0.745	-0.0136	6.5	0.121
4-5 children	0.0039	-1.9	0.721	-0.0195	9.2	0.04
6+ children	0.001	-0.5	0.717	-0.0175	8.2	0.096
***Wealth index***						
Poorest	1.0			1.0		
Poorer	0.0041	-1	0.745	-0.0121	5.7	0.111
Middle	-0.0001	0.05	0.792	-0.004	1.9	0.588
Richer	-0.0002	0.1	0.714	0.0035	-1.6	0.644
Richest	-0.0001	0.03	0.919	-0.0127	6	0.865
***Decision on respondent’s health***						
Respondent alone	1.0			1.0		
Respondent & partner	0.0136	-6.4	0.759	-0.0006	0.3	0.898
Partner alone	0.0042	-2	0.738	-0.0126	5.9	0.21
Someone else	-0.0028	1.3	0.743	0.0002	-0.1	0.96
***Desire of last pregnancy***						
Wanted then	1.0			1.0		
Wanted later	0.0022	-1.1	0.712	-0.0122	5.7	0.028
Wanted no more	0.0004	-0.2	0.775	0.026	-1.2	0.41
***First ANC initiated***						
Later than 1^st^ trimester	100			1.0		
Within 1^st^ trimester	-0.0016	0.7	0.352	-0.0188	-8.7	<0.001
***Distance to Health facility***						
Big problem	1.0			1.0		
Not a big problem	-0.0034	1.6	0.692	-0.0086	4.1	0.58
***Frequency of watching TV***						
Not at all	1.0			1.0		
Less than once a week	0.0004	-0.02	0.759	-0.0001	-0.01	0.984
At least once a week	0.0040	1.9	0.706	0.012	-5.7	0.013
***Constant***				-0.1929	60.8	0.004
***Total***		4.2	0.352		95.8	<0.001

The proportion of women attaining adequate ANC increased from 43% in 2010 to 51% in 2015/16. The slight increase was attributed to 33.8% of changes due to the coefficients and 66.2% due to the changes in the population characteristics. These changes were statistically significant with a *p*-value of <0.001. The increase in the proportion of women who initiated ANC during the first trimester contributed 50.5% to the increase observed in 2010 to 2015/16 surveys. This was statistically significant at a *p*-value of <0.001. In the contributions due to differences in coefficients, the southwest highlands contributed 21.4% to the overall increase (Table 4).

**Table 4:** Decomposition of changes in adequate antenatal care visits 2010 to 2016

Characteristics	Differences in population structure (E)			Differences in coefficients (C)		
	Coefficient	%	***p***-value	Coefficient	%	***p***-value
***Age at delivery (in years)***						
15-19	1.0			1.0		
20-24	-0.0014	-1.7	0.138	-0.010	-12.2	0.209
25-29	-0.0002	-0.2	0.252	-0.009	-11.4	0.255
30-34	0.0002	0.2	0.009	-0.011	-13.1	0.116
35+	0.001	1.2	0.006	-0.001	-1.6	0.875
***Zones***						
Western zone	1.0			1.0		
Northern zone	-0.0005	-6.2	<0.001	0.0013	1.6	0.785
Central zone	-0.0006	-0.8	<0.001	0.0080	9.9	0.113
Southern Highlands	-0.0028	-3.5	0.006	0.0030	3.7	0.385
Southern zone	-0.0017	-2.2	0.004	0.0024	2.9	0.374
Southwest Highlands	-0.0016	-0.2	0.013	0.0173	21.4	0.001
Lake Zone	0.0026	3.2	<0.001	0.0126	15.6	0.239
Eastern zone	0.0108	13.4	<0.001	0.0053	6.6	0.303
Zanzibar	-0.0003	-0.4	<0.001	0.0011	1.4	0.333
***Highest level of education***						
No formal education	1.0			1.0		
Primary education	-0.0011	-1.3	0.324	-0.014	-17.6	0.356
Secondary and above	0.0063	7.1	0.073	-0.002	-2.4	0.367
***Parity***						
1 child	1.0			1.0		
2-3 children	0.0003	0.4	0.009	-0.078	-9.6	0.378
4-5 children	0.0025	3.1	0.001	-0.008	-0.2	0.488
6+ children	0.0023	2.8	<0.001	-0.004	-4.8	0.866
***Wealth index***						
Poorest	1.0			1.0		
Poorer	-0.0003	-0.4	0.645	0.0051	6.3	0.419
Middle	-0.0008	-0.9	0.3	0.0017	2.1	0.761
Richer	-0.0022	-2.7	<0.001	0.0044	5.5	0.428
Richest	0.0028	3.4	0.018	0.0087	10.7	0.122
***Decision on respondent’s health***						
Respondent alone	1.0			1.0		
Respondent & partner	0.0042	5.2	0.255	0.0177	21.9	0.079
Partner alone	0.0057	7.1	0.125	0.0066	8.2	0.508
Someone else	-0.0008	-0.1	0.951	0.0005	0.6	0.728
***Desire of last pregnancy***						
Wanted then	1.0			1.0		
Wanted later	-0.003	-3.7	0.024	0.007	8.7	0.102
Wanted no more	-0.001	-0.1	0.483	-0.0017	-2.1	0.412
***First ANC initiated***						
Later than 1^st^ trimester	1.0			1.0		
Within 1^st^ trimester	0.0408	50.5	<0.001	-0.0016	-2.0	0.459
***Frequency of watching TV***						
Not at all	1.0			1.0		
Less than once a week	0.0007	-0.9	0.821	-0.0016	-1.9	0.542
At least once a week	0.0024	2.9	0.122	-0.0071	-8.8	0.062
***Constant***				-0.0045	-5.5	0.947
***Total***		66.2	<0.001		33.8	0.004

## Discussion

The study findings for all the three surveys suggest that women who had their first ANC visit within the first 12 weeks of pregnancy were more likely to achieve adequate ANC visits. These findings are consistent with other studies done in Tanzania, Peru, Cambodia, Cameroon, Senegal, Uganda and Nepal [24, 25]. This similarity can be explained by various interventions that have been conducted in the mentioned countries on ANC utilization as well as early initiation of ANC visits among pregnant women. This positive association between early ANC initiation and adequate ANC visits has also been reported in many other literatures.

The decomposition analysis suggests that changes in population structure and the effects contributed to the variations in adequate ANC visits overtime. Furthermore, the Tanzania Service Provision Assessment Survey reported on differentials in the quality and availability of health care services offered across regions [26]. In some ways, this can explain the decline observed in the 2004/05 and 2010 surveys. Also, time interval between the two surveys which was a transitional stage in maternal health care as Tanzania adopted FANC in 2002, and challenges in the rollout of the new intervention. Although, we cannot overlook the role of quality of ANC services offered, as it has been documented that poor quality could negatively affect ANC attendance. This may have contributed to the decline observed, although this study was unable to address this [11, 25, 27, 28].

For the 2010 and 2015-16 surveys, the first ANC within the first trimester attributed 50.5% of the increase in the proportion of women wo attained adequate ANC visits to differences due to population structure. Efforts to ensure Tanzania reached the MDG 4 and 5 by 2015 and “Wazazi Nipendeni campaign” in 2012 could explain the increase in adequate ANC attendance in 2010 and 2016 [14, 16]. For the southwest highlands, the increase in the proportion of pregnant women with adequate ANC visits could be attributed to the Wazazi na Mwana campaign in 2 councils in Rukwa one of the regions included in the zone [29, 30]. While it is not possible to directly attribute the impact of these campaigns, they likely played a part in the observed increase in early ANC initiation, which is a contributing factor to adequate ANC attendance. Strengthened and focused efforts are needed where early ANC initiation  and subsequent adequate ANC attendance remain sub-optimal. So, a need to focus on other regions in Tanzania to promote early ANC initiation and subsequently lead to an increase in the number of women attaining adequate ANC.

## Conclusion

The results of this study indicate that adequate ANC attendance has been declining from 2004 to 2010 but a gradual increase has been observed in 2016. ANC initiation within the first twelve weeks of pregnancy has greatly contributed to the recent observed increased proportion of pregnant women who attained four or more ANC visits in Tanzania.

### Study limitation and strength

The study has successfully identified factors associated with changes in adequate ANC visits among pregnant women in Tanzania. With this, it is possible to reallocate the limited resources in Tanzania to focus on the factors that have shown to have a great contribution and influence on attaining adequate ANC visits among pregnant women in Tanzania. We have used nationally representative data which makes the study findings generalizable to the entire nation.

Data on the quality of ANC service was not analyzed in this study, we failed to establish its effect on adequate ANC attendance. Also, the analysis did not include biomedical data on HIV status which could have overestimated the number of ANC visits as HIV-positive women attend ANC on monthly basis. Also, the study is prone to social desirability bias as the women are aware of the recommended minimum ANC visits, which could have overestimated the effects. This study is a Crossectional study, no temporal relationship can be established.

### Recommendation

Basing on the findings obtained, we would recommend the MOHCDGEC and implementing partners to put more effort on promoting the first ANC visit to be initiated within the first twelve weeks of pregnancy. This should be done hand in hand with providing mass education on the importance of ANC visits and why a pregnant women should adhere to the ANC comprehensive package. Intervention should be done at facility and community level. This will enable mothers and the communities to be informed on the pivotal role of ANC services as far as the safety of the mother and child is concerned and ensure continuous support from their families. Regional focused interventions such as ‘Wazazi na Mwana Campaign’ should be rolled out in regions with low uptake of ANC services. Further research to assess the quality of ANC services offered as it may have contributed to the changes observed and sub-optimal ANC attendance while we have a 98% coverage of at least one ANC visit among pregnant women in Tanzania.

## Data Availability

Data and material will be available upon request from the corresponding author with authorization form demographic and health survey program, measure DHS.

## Abbreviations

ANC: Antenatal care
APR: Adjusted Prevalence Ratios
FANC: Focused Antenatal Care
LBW: Low Birth Weight
MDG: Millennium Development Goals
MMR: Maternal Mortality Ratio
SDG: Sustainable Development Goals
TDHS: Tanzania Demographic and Health Survey
WHO: World Health Organization
